# Scalable Continuous Manufacturing Process of Stereocomplex PLA by Twin-Screw Extrusion

**DOI:** 10.3390/polym15040922

**Published:** 2023-02-12

**Authors:** Mohammed Alhaj, Ramani Narayan

**Affiliations:** Department of Chemical Engineering & Material Science, Michigan State University, East Lansing, MI 48824, USA

**Keywords:** PLA, stereochemistry, stereocomplex, extrusion, crystallinity, melting temperature

## Abstract

A scalable continuous manufacturing method to produce stereocomplex PLA was developed and optimized by melt-blending a 1:1 blend of high molecular weight poly(L-lactide) (PLLA) and high molecular weight poly(D-lactide) (PDLA) in a co-rotating twin-screw extruder. Thermal characteristics of stereocomplex formation were characterized via DSC to identify the optimal temperature profile and time for processing stereocomplex PLA. At the proper temperature window, high stereocomplex formation is achieved as the twin-screw extruder allows for alignment of the chains; this is due to stretching of the polymer chains in the extruder. The extruder processing conditions were optimized and used to produce >95% of stereocomplex PLA conversion (melting peak temperature T_pm_ = 240 °C). ATR-FTIR depicts the formation of stereocomplex crystallites based on the absorption band at 908 cm^−1^ (β helix). The only peaks observed for stereocomplex PLA’s WAXD profile were at 2θ values of 12, 21, and 24°, verifying >99% of stereocomplex formation. The total crystallinity of stereocomplex PLA ranges from 56 to 64%. A significant improvement in the tensile behavior was observed in comparison to the homopolymers, resulting in a polymer of high strength and toughness. These results lead us to propose stereocomplex PLA as a potential additive/fiber that can reinforce the material properties of neat PLA.

## 1. Introduction

**Figure 1:**
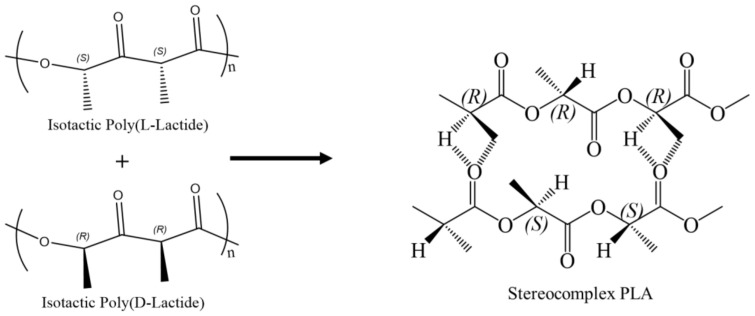
Enantiomeric PLA homopolymers blend to form stereocomplex PLA.

Polylactide (PLA) is extensively used in medical applications (drug delivery, scaffolds, bone growth, and tissue regeneration) and recently in industrial products such as packaging and 3D printing [1,2,3,4,5]. Studies have shown that stereochemistry can play a role in the material properties of polylactide (PLA). Increasing the D-lactide content or meso-lactide content when copolymerizing with L-lactide would lead to a reduction in the polymer’s melting point and crystallinity [6,7]. However, the same behavior cannot be said when physically mixing poly(L-lactide) (PLLA) and poly(D-Lactide) (PDLA) as opposed to copolymerizing L-lactide and D-lactide. When the interaction between polymers of varying configurations and/or tacticities dominates over the one between polymers with similar configurations and/or tacticities, a stereoselective interaction between the polymers takes place. Such association is defined as stereocomplex formation aka stereocomplexation [8,9,10,11,12,13,14]. The stereocomplexation of PLA may occur in solution, in the melt state, or during polymerization [15]. In the melt state, PLLA and PDLA chains begin to form α-domains during a cold crystallization process. Additional heating leads to the melting of α-domains and the permanent rearrangement of the chain structure [16]. In solution, however, stereocomplex crystals experience improved solvent stability compared to homocrystals. This leads to the self-assembly of PLLA and PDLA chains into stereocomplex crystals in organic solvents, which include chlorinated solvents (dichloromethane, chloroform) and tetrahydrofuran [17,18]. Based on several studies, researchers have deduced that the ratio of stereocomplex crystallites to homocrystallites is mainly affected by the molecular weights of the homopolymers, the optical purities of the homopolymers, the steric hindrance of the pendant chain, backbone rigidity, tacticity, and the mixing ratio [19,20,21]. Studies have also shown that PLA homopolymers of high melting temperatures and crystallinities are favorable for stereocomplex crystallite formation. Moreover, when the crystallinities of the stereocomplex PLA of two different MW blends (low/high) are similar, the blend with a higher molecular weight facilitates a higher stereocomplex melting temperature [22,23]. By blending PLLA and PDLA at a 1:1 ratio above their melting point, one can form a stereocomplex with increased crystallinity and a melting point that is ~50 °C higher than the PLA homopolymers. The mechanism behind the observed increase in melting temperature is related to the packing of the stereocomplex helices, which are stabilized by strong van der Waals interactions and hydrogen bonding [24,25,26]. Figure 1 displays the stereochemical structure of PLLA and PDLA as they blend to form a stereocomplex structure based on hydrogen bonding between the methyl hydrogen of one PLA compound and the carbonyl oxygen of its respective enantiomeric PLA compound.

The resulting conformations offer favorable positions for the polymer loops during the crystallization process. This process significantly reduces the induction period, leading to the formation of highly dense spherulites [27]. Given the advantageous crystallization process, researchers have observed significant improvement in the PLA barrier properties [28,29,30]. One study found that the water vapor permeability values of solution-casted stereocomplex-based films were significantly reduced (~14–23%) compared to those of the PLLA/PDLA homopolymers [31]. Varol et al. conducted a more detailed investigation into the effect of PLA stereocomplexation on the transport properties for a variety of permeants (water, nitrogen, oxygen, and carbon dioxide). A significant barrier improvement of ~70% was observed for the resulting stereocomplex compared to the corresponding PLA homopolymers with similar crystallinity [28].

In order to improve the thermal/mechanical performance as well as the hydrolysis resistance of PLA, researchers have conducted many studies on PLA stereocomplexation using a variety of additives, nucleating agents, rheometers, and/or solvent-based techniques [32,33,34,35,36,37,38,39,40]. Table 1 summarizes the results in comparison to our technique. Samsuri et al. used a solution casting technique combining synthesized PLLA and PDLA in the presence of stereocomplex PLA particles of varying particle sizes for the enhanced formation of stereocomplex crystallites. With a stereocomplex PLA (SC PLA) particle content ranging from 3 to 20%, the researchers achieved a stereocomplex formation of up to 49% and a crystallinity up to 45%, with a melting peak temperature up to 226 °C [41]. Qi et al. conducted a similar solution blending method to produce stereocomplex PLA containing glucose groups (sc-PLAG); the glucose content ranged from 0.5 to 5%. Optimal thermal properties were observed at 2% glucose, displaying >99.99% of stereocomplexation, a melting peak temperature for a stereocomplex of ~206 °C, and a crystallinity of ~35%. It was concluded that glucose groups heavily influenced the stereocomplexation process by causing heterogeneous nucleation, promoting amphiphilic self-assembly, and affecting the ordered arrangement of PLA chains [42].

More recently, triblock terpolymers of polystyrene-b-stereocomplex PLA-b-poly(2-vinylpyridine) (PS-b-SC-b-P2VP) were synthesized and characterized by solution blending block copolymers of PS-*b*-PDLA and P2VP-*b*-PLLA. At the highest molecular weight (~11 kDa) of the PLA homopolymer samples, >99.9% of stereocomplexation occurred, with a stereocomplex melting temperature of ~230–70 °C higher than the respective PLA homopolymers- and a crystallinity of ~39% [43]. The increase in the thermal properties and crystallinity may be attributed to the increasing molecular weight of the PLA blocks. Studies have also shown that stereocomplex formation and material properties could be further promoted via the use of cellulose nanofibers (CNFs) [44,45,46]. Ren et al. prepared a PLLA/PDLA blend at different CNF concentrations by melt compounding CNF-based PLA nanocomposites, followed by solution blending the composite with a 1:1 blend of PLLA and PDLA. At 3% CNF, ~70% of stereocomplex PLA resulted, with a melting peak temperature of ~219 °C and a total crystallinity of ~43%. Heating the samples at a nonisothermal crystallization temperature of 190 °C, followed by a second heating scan to 250 °C, displayed a further increase in stereocomplex formation, with the maximum crystallinity at ~51% and a melting peak temperature of ~220 °C [46]. This enhanced formation of SC crystallites may be due to the interaction of the surface hydroxyl groups of the CNFs with the PLA enantiomers.

To avoid the thermal degradation of PLA chains and suppress the growth of PLA homocrystallites due to high temperature processing, Gao et al. presented an alternative route to rapidly prepare an exclusive PLA stereocomplex induced by shear stress at low temperature (190 °C) extrusion in a rheometer. Using poly(butylene adipate-co-terephthalate) (PBAT) (0–50%), PLA crystallization was accelerated and processability of the PLA stereocomplex at low temperature was improved due to the decrease in the melt flow’s activation energy. At 10% PBAT content, full stereocomplexation occurred, and the crystallinity and stereocomplex melting temperature both increased to 56.7% and 228 °C, respectively [47]. These results justify that PBAT can be used as a possible additive to continuously produce exclusive stereocomplex PLA at low processing temperatures.

Su et al. presented a solventless technique, with no additives, via micro-extrusion in a rheometer to produce stereocomplex PLA from high molecular weight PLLA and PDLA; >99.99% of stereocomplexation occurred, with a melting peak temperature of ~230 °C and a crystallinity of ~44% [34]. Korber et al. tried to scale up this process in a pilot-scale co-rotating twin screw extruder (L/D = 40). Without additives/nucleators, only ~25% of stereocomplex formation occurred, with a stereocomplex melting peak temperature of ~220 °C. Only the use of two additives (aluminum complex with phosphoric ester NA-21 and NA-21 + talc) resulted in >99.99% of stereocomplex formation, but the melting peak temperature was still ~220 °C and the crystallinity was ~40% [38]. The most successful results in achieving exclusive stereocomplexation with excellent thermal properties originated from a recent study in Hungary. PLLA and PDLA were melt blended in a counter-rotating twin-screw extruder (L/D = 44) at a temperature range of 220–235 °C, then melt blown in a single-screw laboratory extruder (L/D = 24) at a temperature of 240 °C. The results showed >99% of stereocomplex fibers with a crystallinity of ~55% and a stereocomplex melting temperature of ~220 °C. The large extent of stereocomplex formation was due to the high shearing force caused by high-velocity air from melt blowing, leading to improved stereoselective interaction between the PLLA and PDLA chains [48]. However, this was a two-step process to produce stereocomplex PLA and resulted in a lower melting temperature, most likely due to the molecular weights of the homopolymers—PLLA (M_n_ = 72 kDa) and PDLA (M_n_ = 50 kDa) [35,38]. It is clear that no scalable, solventless, additive-free one-step process to produce exclusive stereocomplex PLA using high molecular weight PLA (M_n_ >90 kDa) has been reported in journal articles.

**Table 1:** Recent studies on stereocomplex PLA.

Group	Method	*f_sc_* (%)	T_pm,sc_ (°C)	*X_c_* (%)
Samsuri [41]	Solution casting with SC PLA particles	60	220	23
Arkanji [43]	Solution blending of PS-b-PDLA and P2VP-b-PLLA	>99	231	39
Baimark [49]	Solution blending/precipitation of PLLA/PDLA (low MW)	>99	219	60
Su [34]	Rheometer (190–220 °C)	>99	230	44
Korber [38]	Co-rotating twin screw extrusion (180–240 °C)	25	220	10
Kara [48]	Counter-rotating twin screw extruder (220–235 °C) ⟶ single screw lab extruder (240 °C)	>99	220	55
Alhaj	Co-rotating twin screw extruder (180–220 °C)	95	240	58

Herein, we describe a scalable, solventless, additive-free continuous manufacturing method via twin-screw extrusion to produce an exclusive stereocomplex PLA only from 1:1 blending of high molecular weight (M_n_ >90 kDa) PLLA and PDLA homopolymer in a co-rotating twin-screw extruder (L/D = 40). The thermal characteristics of stereocomplex formation were first analyzed to optimize the processing parameters for achieving the highest stereocomplex formation and the optimal thermal properties (crystallinity, melt temperature, etc.). Stereocomplex PLA was then continuously produced as pellets in a co-rotating twin-screw extruder (L/D = 40 and screw diameter = 27 mm). The final product may then be used in additive application development to produce molecular composites of PLLA/SC PLA. We conclude this paper with improvements and suggestions for the production and application of stereocomplex PLA.

## 2. Materials and Methods

### 2.1. Materials

The >99.99% neat PLLA (L175) and PDLA (D120) were obtained from TotalEnergies Corbion (The Netherlands) in the form of pellets, and dried for 24 h at 45 °C. Table 2 summarizes their material properties. The optical purity was confirmed via polarimetry. The tensile strength (σ_ult_) and elastic modulus (*E*) were reported in the data from TotalEnergies [50,51]. PLLA and PDLA were pre-mixed at a 50/50 ratio and then further dried for another 24 h. No reagents were used. The glass transition temperature (T_g_) and melting peak temperature (T_pm_) were identified using ASTM D3418 and ISO 11357-3 [52,53].

### 2.2. Thermal Characteristics of Stereocomplex Formation

**Figure 2:**
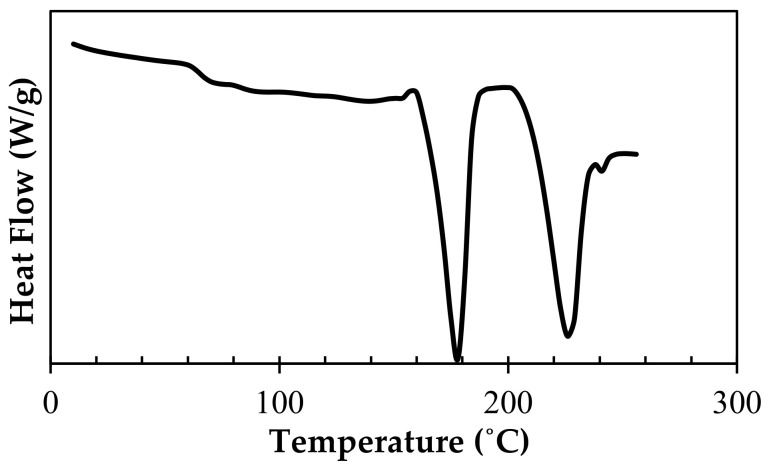
The first DSC thermogram of pellets comprising 50% stereocomplex crystallites/50% PLA homocrystallites based on the melting enthalpy calculations.

The optimal processing conditions for stereocomplexation were determined via DSC. A pellet comprising 50% stereocomplex PLA/50% PLA homopolymer was first developed and characterized via DSC, as shown in Figure 2. The fractional stereocomplex crystallites *f_sc_* and homocrystallites *f_hc_* were calculated based on melting enthalpy integration:

$$f_{sc}=\frac{X_{sc}}{X_{sc}+X_{hc}}$$

$$f_{hc}=\frac{X_{hc}}{X_{sc}+X_{hc}}=1-f_{sc}$$

where $X_{sc}$ and $X_{hc}$ are the experimental crystallinities of the stereocomplex crystallites and homocrystallites, respectively [54].

The 50% stereocomplex PLA and 50% PLA homocrystals were confirmed based on the melting peaks at 225 °C and 178 °C, respectively. It was assumed that the PLA homocrystals were an unreacted blend of PLLA and PDLA. The conversion of this unreacted blend of PLA homocrystallites to stereocomplex crystallites was characterized based on the time and temperature. The temperature was first equilibrated to 180/190/200/210/220/230 °C; these temperatures were chosen as they are at or above the melting peak temperature of PLA (~180 °C). It is required to process PLLA and PDLA in the liquid state to form stereocomplex PLA in order to allow for freedom of motion in the molecules [8]. The temperature was then held constant at 1/2/3 min, as the processing time in the extruder is no more than 3 min. Samples were then cooled to 0 °C at 10 °C/min, then held isothermally for 2 min. A heating scan was finally run at 10 °C/min to 250 °C. This heating scan was then analyzed to calculate the fractional stereocomplex crystallites based on melting enthalpy integration. The stereocomplex formation was plotted at different times and temperatures to model the kinetics.

### 2.3. Twin-Screw Extrusion of Stereocomplex PLA

The procedure thereof involves a scalable technique to continuously produce SC PLA in a co-rotating twin-screw extruder (L/D = 40 and screw diameter = 27 mm). High molecular weight PLLA (M_n_ = 103 kDa) and PDLA (M_n_ = 92 kDa) were blended in a 1:1 ratio with no additives into the feeding zone of a co-rotating twin-screw extruder type ZSE 27 HP–PH from Leistritz (Nürnberg, Germany).

**Figure 3:**
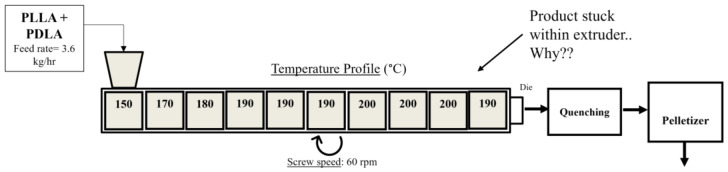
Processing conditions for the first trial run of stereocomplex PLA in a co-rotating twin-screw extruder type ZSE 27 HP–PH from Leistritz.

The heating system was divided into 10 heating zones. The temperature profile was carefully chosen according to previous studies on the thermal behavior of SC PLA during processing. As seen in Figure 3, it was discovered that processing a 1:1 blend of PLLA and PDLA at temperatures lower (~190–200 °C) than the melting temperature of stereocomplex PLA (~230–240 °C) would lead to solidification of the product within the extruder. This is especially true when setting such temperatures in the first few heating zones of the extruder. The reason for this behavior is due to the formation of SC PLA within the extruder as the temperatures of the heating zones are not high enough to flow the product through the extruder.

**Figure 4:**
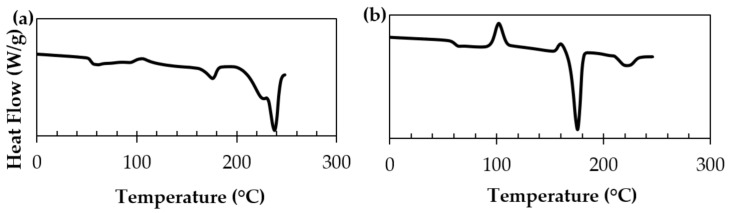
The DSC thermograms on stereocomplex PLA (**a**) First heating scan—stereocomplex crystallite formation (**b**) Second heating scan—thermal dissociation of stereocomplex crystallites to homocrystallites.

Differential scanning calorimetry (DSC) studies (Figure 4) also showed that processing the PLLA/PDLA blend at temperatures at or above the melting point of stereocomplex PLA led to thermal dissociation of the stereocomplex. This means that the stereocomplex PLA product reverts to its respective PLLA and PDLA homopolymers. The reason behind this behavior is most likely due to the chain slipping and breakage of the stereocomplex crystallites due to melting. The product stems from a physical blend, not a chemical reaction, of two polymers. This results in thermal instability at or above its melting temperature; it is an entropy driven process. In the melt state, the PLLA and PDLA units that once formed a stereocomplex are now free to move about in any order compared to the solid state; there is an increase in the molecular motion of the system, and thus, an increase in entropy. This increased freedom of motion results in a higher variation in possible locations for the molecules.

Taking these factors into consideration, one must carefully consider the temperature conditions to process SC PLA. A temperature profile of 160/170/180/180/180/210/220/220/220/230 (Heating Zone 1 to Die Temperature) was chosen based on the DSC results on the stereocomplexation kinetics (See Section 3.1). This profile focused on heating the beginning of the extruder barrel at temperatures high enough (up to 180 °C) to flow the PLLA/PDLA blend but low enough to prevent excess stereocomplex formation too early. The end of the barrel is then heated at higher temperatures (~210–220 °C) to accelerate stereocomplexation within the last minute of the process. This method prevents solidification and thermal dissociation of the final product, allowing for the continuous manufacturing of SC PLA.

**Figure 5:**
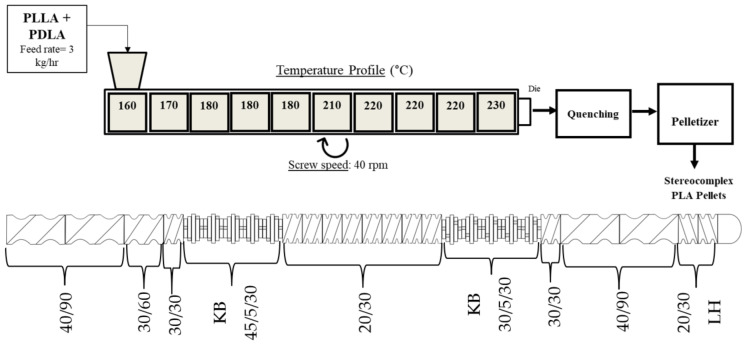
Processing conditions for the new trial run of stereocomplex PLA in a co-rotating twin-screw extruder type ZSE 27 HP–PH from Leistritz.

Figure 5 displays the final processing conditions chosen to extrude stereocomplex PLA. A feed rate of 3 kg/hour and screw speed of 40 rpm was chosen. The screw configuration was constructed to allow for optimal mixing and transport of the PLLA and PDLA homopolymers. Kneading elements were placed in the first and second half of the barrel in between the conveying elements. This was to allow for mixing in the first and last minute of processing, while enabling material transport throughout the extruder. The residence time was 2.5 min until the product was extruded out of a 5 mm diameter strand die, then quenched in a cold-water bath and pelletized. Samples were collected at different time intervals (5, 10, 20, 40 min) to analyze the dispersity of the stereocomplex. Samples were then dried in an oven at 55 °C for 24 h.

### 2.4. Injection Molding of Stereocomplex PLA

SC PLA and PLLA (L175) samples were injection molded into tensile bars on a NEGRI BOSSI V55-200 injection molding machine. Injection molding of the tensile bars was conducted at injection and mold temperatures of 220 °C and 55 °C, respectively; dwell time was 10 s. Samples were then annealed at 100 °C for 2 h.

### 2.5. Characterization and Analysis

PLA stereocomplexation was confirmed using a Shimadzu IRAffinity-1. The changes in the conformation of PLA chains were observed using attenuated total reflectance-Fourier transform infrared spectroscopy (ATR-FTIR). The α helix (wavenumber 921 cm^−1^), which is characteristic to PLLA and PDLA, was transformed into a more compact β helix (wavenumber 908 cm^−1^) [55]. The SC PLA spectrum was analyzed and compared to neat PLLA (L175).

The crystal structure of the samples was determined by wide angle X-ray diffraction (WAXD). The spectra were recorded with a Rigaku Smartlab X-ray Diffractometer at room temperature using CuKα radiation at 40 kV and 44 mA. The specimens were scanned in the scanning range of 5–30° at a scan speed of 3°/min. Spectra were analyzed using GSAS-II [56]. *f_hc_* and *f*_sc_ are the fractional amounts of homocrystallites and stereocomplex crystallites, respectively, developed during processing under non-isothermal conditions; these can be calculated using the following equations:

$$\;f_{sc}=\frac{A_{sc}}{A_{sc}+A_{hc}}$$

$$f_{hc}=1-f_{sc}=\frac{A_{hc}}{A_{sc}+A_{hc}}$$

where *A_sc_* and *A_hc_* are the integrated area under the respective curves for stereocomplex crystallites and homocrystallites, respectively. The total crystallinity was also calculated using the following equation:

$$X_{c}=\frac{A_{sc}+A_{hc}}{A_{sc}+A_{hc}+A_{amph}}$$

where *A_amph_* is the integrated area under the curve for the amorphous phase.

These calculations were supplemented via DSC using a TA Instruments DSC Q20. The thermal properties were also characterized. The temperature was first equilibrated to 0 °C, then the temperature was ramped up to 260 °C at a heating rate of 10 °C/ min. A second heating scan was not run because, above its melting temperature (~240 °C), stereocomplex PLA thermally dissociates into its constituent homopolymers; this is due to molecular diffusion [57,58,59]. *f_sc_* and *f_hc_* can be calculated using the following equations:

$$f_{sc}=\frac{X_{sc}}{X_{sc}+X_{hc}}$$

$$f_{hc}=\frac{X_{hc}}{X_{sc}+X_{hc}}=1-f_{sc}$$

where $X_{sc}$ and $X_{hc}$ are the crystallinities of the stereocomplex crystallites and homocrystallites, respectively [54]. The total crystallinity can then be calculated via the following formula:

$$X_{c}=\frac{∆H_{m,sc}+∆H_{m,hc}}{f_{sc}*∆H_{m,sc}^{0}+f_{hc}*∆H_{m,hc}^{0}}$$

where $∆H_{m,sc}^{0}$ and $∆H_{m,hc}^{0}$ are the theoretical enthalpy values for a single crystal of β-form stereocomplex (142 J/g) and a single crystal of α-form homocrystallite (93 J/g) [60,61].

Tensile testing of the injection molded samples was conducted according to ASTM D638 in an Instron model 5565-P6021, at a crosshead speed of 1 mm/min [62]. The yield strength, ultimate tensile strength, strain at break, plastic strain, elastic modulus, toughness, and modulus of resilience were statistically measured for five samples at 25 °C and a relative humidity of 50 ± 5%.

The thermal decomposition temperature and percent weight loss were quantified via thermogravimetric analysis (TGA), specifically the TGA Q50. About 10 mg of the sample was heated from 25 to 550 °C at 20 °C/min.

## 3. Results and Discussion

We developed a scalable continuous manufacturing process via twin-screw extrusion to produce >95% SC PLA with a melting peak temperature of ~240 °C; no additives, nucleators, or reagents were used, aside from high molecular weight neat PLLA and PDLA. At the proper temperature window, high stereocomplex formation can be achieved as the twin-screw extruder allows for alignment of the chains; this is due to stretching of the polymer chains in the extruder. Compared to traditional processing methods (i.e., single-screw extrusion), we believe that the twin-screw extruder provides higher process productivity/capability, higher melting capabilities, and much higher mixing efficiency, which is imperative in PLA stereocomplexation [63,64,65]. In the next sections, we will delve further into the results of this process.

### 3.1. Thermal Characteristics of Stereocomplex Formation

**Figure 6:**
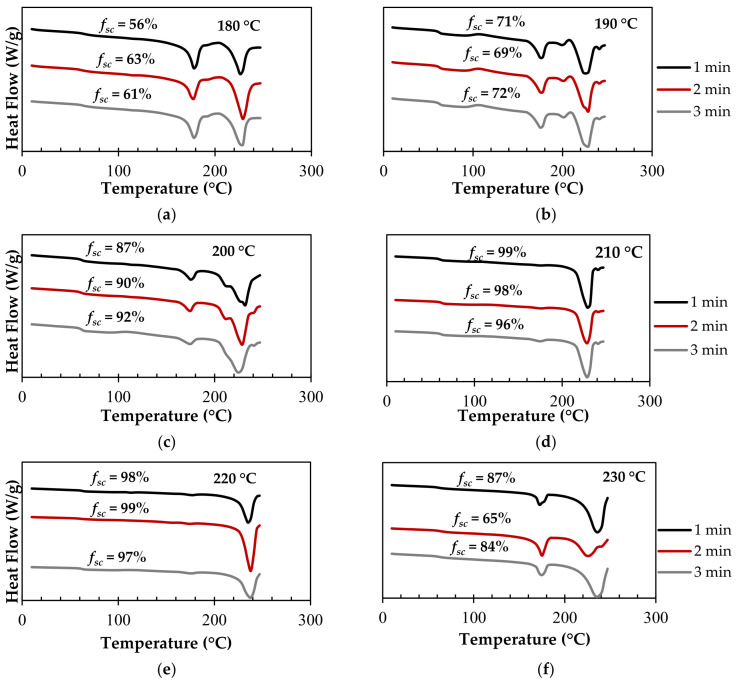
The DSC thermograms displaying stereocomplex formation after the sample was held isothermally for 1–3 min at temperatures of (**a**) 180 °C, (**b**) 190 °C, (**c**) 200 °C, (**d**) 210 °C, (**e**) 220 °C, (**f**) 230 °C.

In order to optimize the processing conditions for stereocomplexation, DSC was run at different times and temperatures for samples comprising 50% stereocomplex PLA crystallites/50% PLA homocrystallites. Heat flow vs. temperature graphs were plotted at different times; each graph represents the isothermal processing temperature the sample is held at during the run. The results are shown in Figure 6.

At 180 °C (Figure 6a), ~60% SC PLA resulted, regardless of the processing time. This is equivalent to the melting temperature of the PLA homopolymer. Considering the original sample comprised 50% stereocomplex crystallites, the data show that 10% stereocomplexation will occur at the melting temperature of PLA.

When the sample was held isothermally at 190 °C (Figure 6b), ~70% SC PLA was calculated, resulting in 20% of stereocomplex formation compared to the original sample. The processing time did not make any significant changes in this condition either.

Figure 6c displays the isothermal processing temperature at 200 °C. Compared to the previous two runs at 180 and 190 °C, the fractional stereocomplex crystallites herein were calculated to be ~90%. This is equivalent to 40% of stereocomplexation occurring at this processing temperature, which is 2× the amount formed at 190 °C and 4× the amount formed at 180 °C.

Figure 6d,e represent the 210 and 220 °C isothermal processing temperatures, respectively. It was concluded that once the sample was heated to 210 °C, almost 100% of SC PLA was formed. However, increasing the temperature further to 230 °C (Figure 6f) resulted in a decrease in the stereocomplex crystallites compared to Figure 6d,e. This phenomenon is due to thermal dissociation, in which the freedom of motion in the PLLA and PDLA chains of the stereocomplex PLA increases. At 230 °C, the SC PLA sample is in the melt state, resulting in an increase in the molecular motion of the polymer chains, and thus an increase in entropy. Bao et al. explained that the formation of stereocomplex crystallites loses efficiency at higher processing temperatures close to melting, most likely due to an increase in the chain mobility being overcome by the significant decrease in nucleation density. At a low degree of supercooling, the stereocomplex crystallization rate decreases [66]. Another more recent study further described this phenomenon as “poisoning by purity”, in which the growth of the pure PLA homopolymer is rejected from the SC ahead of its growth front. They contributed this phenomenon to random compositional fluctuations present in the melt [67].

**Figure 7:**
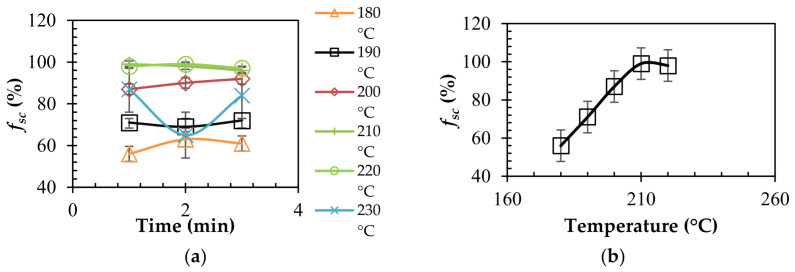
(**a**) Isothermal stereocomplexation kinetics. (**b**) Effect of temperature on stereocomplex formation.

We concluded that stereocomplexation is a temperature driven process, and that time has a minimal impact on the process. The phenomenon is almost instantaneous at temperatures above PLA’s melting point. Figure 7a summarizes the isothermal stereocomplexation kinetics of the samples discussed. The results further confirm that stereocomplex PLA strongly depends on the processing temperature of PLLA/PDLA pre-mixtures, and that time does not play a role.

Figure 7b displays the effect of temperature on stereocomplex formation at 2 min; similar results were seen at 1 and 3 min. There was a linear relationship between the temperature and stereocomplex formation until 210 °C, where the curve flattened, since ~99% of stereocomplexation occurred. This led us to further conclude that processing temperatures at 210–220 °C will lead to the highest conversion of stereocomplex PLA, while preventing thermal dissociation of the sample.

### 3.2. Confirmation of Stereocomplex Formation by FTIR

**Figure 8:**
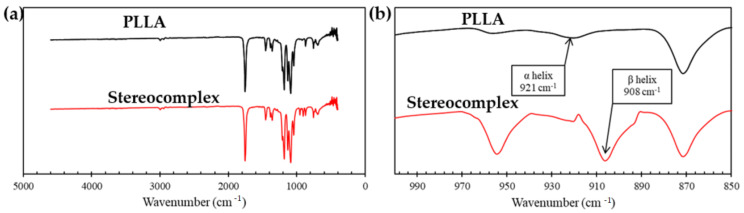
(**a**) FTIR spectra of the PLLA (black line) and stereocomplex PLA (red line). (**b**) FTIR spectra displaying characteristic absorption bands. The spectra displayed involves the stereocomplex PLA collection time at 20 min.

Stereocomplexation was confirmed via ATR-FTIR in comparison to neat PLLA. Figure 8a shows the ATR-FTIR spectrum of the PLA stereocomplex compared with the spectrum of the PLLA enantiomer. Regions of wavelength that involved characteristic changes were observed at 970–850 cm^−1^. These changes in conformational PLA chains represent skeletal stretching vibration of the α and β helices. Figure 8b clearly depicts the α helix of the PLLA chain transformed to a more compact β helix in the stereocomplex PLA. Specifically, the absorption band at 908 cm^−1^ represents the presence of stereocomplexation. The formation of stereocomplex crystallites is the ultimate result of the stereoselective interaction between opposite enantiomeric PLA chains. It has been suggested that van der Waals forces between the carbonyl oxygen and the methyl hydrogen occur during stereocomplexation [24]. However, the establishment of hydrogen bonding between the carbonyl oxygen of one PLA compound and the methyl hydrogen of its respective enantiomeric PLA compound is responsible for the formation of the stereocomplex due to the presence of the opposite helical structure in PLLA and PDLA. Moreover, the structural adjustment of the CH_3_ group occurred prior to that of the C–O–C backbone during the stereocomplexation process [25,68]. The FTIR spectra were the same regardless of the sample collection times (5, 10, 20, 40 min).

### 3.3. Crystal Structure Characterization via WAXD

**Figure 9:**
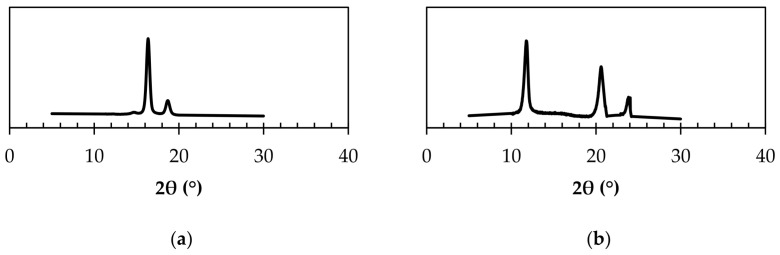
WAXD profiles of (**a**) pure PLLA and (**b**) stereocomplex PLA (50/50 L/D).

The crystal structure, total crystallinity, and stereocomplex formation were analyzed and compared between the neat PLLA and the produced stereocomplex PLA sample using wide angle X-ray diffraction (WAXD). Figure 9a displays the peaks at 2θ values of 15, 17, and 19°, which represent the α form of PLLA crystallized in a pseudo-orthorhombic unit cell with the dimensions *a* = 1.07 nm, *b* = 0.595 nm, and *c* = 2.78 nm; the cell is composed of two 10_3_ polymeric helices [69,70,71,72]. The crystallinity for the PLLA sample was estimated to be ~45%. For SC PLA (Figure 9b), the peaks observed occur at 2θ values of 12, 21, and 24° [66].

Similar to previous reports, the spectrum represents stereocomplex PLA crystallized in a triclinic unit cell with the dimensions: *a* = 0.915 nm, *b* = 0.915 nm, and *c* = 0.868 nm; α = 109°, β = 109°, and γ = 110°. Okihara et al. proposed that the PLLA and PDLA chains were packed regularly in a 3_1_ helical conformation. The lattice comprising PLLA and PDLA chains with a 3_1_ helical conformation had the shape of an equilateral triangle, which was proposed by Okihara to form equilateral-triangle-shaped single crystals of the stereocomplex [73].

Compared to the PLLA sample analyzed, the total crystallinity was measured to be ~61%, which was higher than the crystallinity (<50%) of the PLLA and PDLA blended to produce SC PLA. These results indicate that an increase in crystallinity occurred, possibly due to the parallel packing of the PLLA and PDLA chains that occurs during stereocomplexation. The regular packing of the enantiomeric PLA chains possessing 3_1_ conformational helices represents ordered packing of the chains, which can translate to an increase in crystallinity.

Since the only peaks observed for SC PLA’s WAXD profile were at 2θ values of 12, 21, and 24°, it was concluded that >99% of stereocomplex formation occurred during processing of the PLLA/PDLA blends. This confirms the high conversion of stereocomplex PLA resulting from twin-screw extrusion. From these results, it can be concluded that a proper temperature profile is critical for processing exclusive stereocomplex crystallites of high crystallinity, without any additional formation of homocrystallites.

### 3.4. Thermal Characterization via DSC

**Figure 10:**
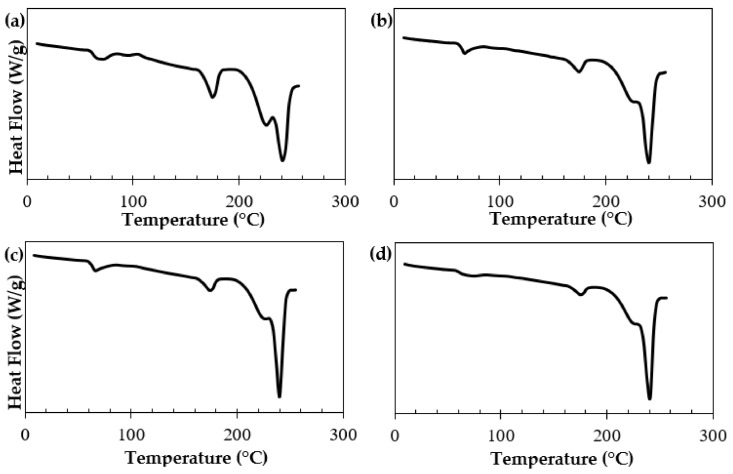
The DSC thermograms of SC PLA at sample collection times of (**a**) 5 min, (**b**) 10 min, (**c**) 20 min, (**d**) 40 min.

The total crystallinity and stereocomplex formation of the extruded samples were quantified using DSC at various sample collection times. It is important to note that stereocomplex crystallites can form or dissociate during melting, which is why WAXD was used to more accurately measure the crystallinity [58,74]. Figure 10 depicts the first DSC thermograms at 5-, 10-, 20-, and 40-min collection times. No additional PLLA, PDLA, or other materials were fed to the extruder during these collection times; these were based on one batch of PLLA/PDLA pre-mixtures.

At 5 min (Figure 10a), ~85% of the PLA stereocomplex crystallites formed, with the remaining 15% composed of PLA homocrystallites. The melting temperature of the stereocomplex was found to be ~241 °C, while the PLA homopolymer melted at 178 °C. Total crystallinity was calculated to be ~64%. A glass transition temperature was also depicted at ~63 °C, most likely from the residual PLA homopolymer.

At 10 min (Figure 10b), a significant increase in stereocomplexation (~92%) was observed. This may be due to stabilization of the melt and the high throughput of the extruder at the beginning of the process. The melting temperature of the stereocomplex remained at ~240 °C, while the PLA homopolymer melted at 175 °C. The total crystallinity was calculated to be ~61%. A 20-min collection time (Figure 10c) only showed a partial increase (~93%) in stereocomplexation. The crystallinity and melting temperature remained the same, indicating stabilization of the melt. At 40 min (Figure 10d), ~95% of the PLA stereocomplex crystallites formed, but the total crystallinity was reduced to ~56%. This contrast in data may be because there was not enough time to cool the melt in the water bath, as the water bath warmed up by 40 min, allowing for slower crystallization.

Table 3 summarizes the DSC results discussed on SC PLA. The results show that stereocomplexation was at its lowest at 5-min collection. This was most likely because there was not enough time for the PLLA/PDLA blend to be dispersed uniformly and stabilize. Over time, however, the blend stabilized and up to 95% of the stereocomplex PLA was continuously produced through the extruder. This is different compared to WAXD’s results on stereocomplex formation—>99%. The 4–5% discrepancy may be attributed to a combination of stereocomplex formation and dissociation in the DSC. From 10 min to 50 min, there were no significant differences in the thermal properties and stereocomplex conversion. We confirmed the uniform dispersity of SC PLA via twin-screw extrusion at >5-min collection time.

After 5 min, the results displayed a fractional stereocomplex formation of 92–95%, total crystallinity of 56–64%, a stereocomplex melting peak temperature of ~240 °C, and a glass transition temperature of 61–65 °C. Compared to the DSC data of its constituent homopolymers (L175 and D120) as seen in Table 2, the stereocomplex product exhibited >60 °C higher T_pm_ and up to a 17% increase in crystallinity. We attributed this significant increase in material properties to the combined effect of processing temperature window and molecular diffusion. In addition, stereocomplex formation may be promoted in the twin-screw extruder due to stretching of the polymer chains. This allows for the further alignment of high molecular weight PLLA and PDLA chains, and may improve the stereoselective interaction between the enantiomers.

### 3.5. Mechanical Properties of Stereocomplex PLA

**Figure 11:**
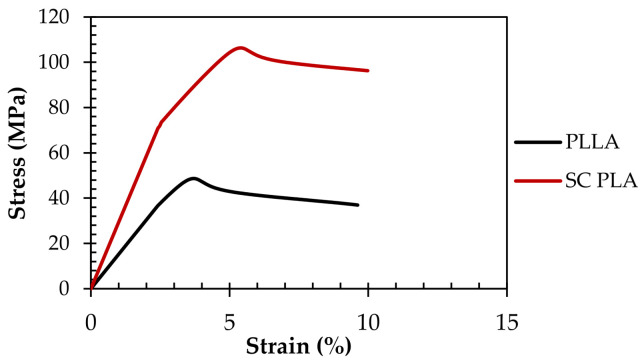
The tensile stress–strain curves of injection molded samples of neat PLLA (L175) and stereocomplex PLA.

Tensile testing was conducted on five injection molded samples of stereocomplex PLA (SC PLA) according to ASTM D638-14 [62]. Results were statistically processed and compared to PLLA (L175) base resin used in the production of SC PLA. Previous studies have reported an improvement in the tensile properties, particularly the elastic modulus and ultimate tensile strength, of stereocomplex PLA compared to its PLA homopolymer counterpart [32,34,38,75]. Theoretically, there should be an increase in the mechanical properties of the polymer as the crystallinity increases, as the densely packed crystallites improve the strength of the polymer, whereas the amorphous regions are responsible for the elastic behavior of the polymer [76,77]. Figure 11 below displays the stress–strain curves for the PLLA and stereocomplex PLA. Data were taken for five samples of each polymer, and the samples closest to the average data are displayed.

As seen in Table 4, a significant increase in the mechanical properties was observed in stereocomplex samples compared to their PLLA base resin. Particularly, the yield strength (σ_y_) and modulus of resilience (*U_R_*) almost doubled when PLA converted to SC PLA. Additionally, the ultimate tensile strength (σ_ult_) of the SC PLA samples was more than two times that of its respective PLLA homopolymer. A 92% increase in the elastic modulus (*E_elastic_*) was also seen from PLLA to SC PLA. In addition, there was a 134% increase in the toughness of the polymer. Almost a 100% increase in the resilience was also observed, which coincides with the increase in toughness. This considerable increase in tensile properties may be strongly dependent on its triclinic crystal structure, allowing the sample to absorb more energy before fracture. This significant improvement in the tensile properties of SC PLA compared to the PLA homopolymer may be related to the intermolecular forces that occur between the PLLA and PDLA chains. As the enantiomers melt and their chains are stretched in the extruder, chain alignment occurs, leading to an increase in stereocomplex formation and crystallinity. This increase in stereocomplex crystallization may align with the increase in the mechanical properties discussed. No significant change in the strain at break (ε_break_) or the plastic strain to failure (ε_p_) was observed, which is expected, as they are more influenced by the amorphous regions than the crystalline domains in semi-crystalline polymers [76,77]. These results provide insights into the possibility of using SC PLA as a fiber to reinforce the mechanical and thermal properties of PLA.

**Figure 12:**
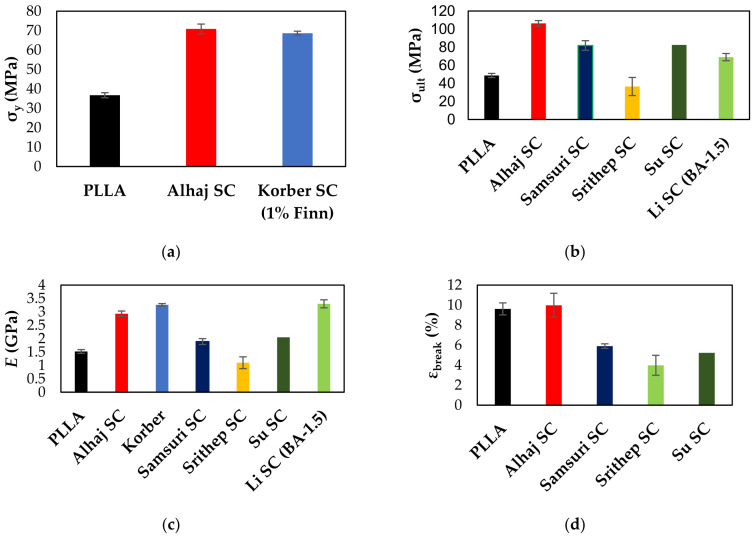
Comparison of the tensile behavior of extruded stereocomplex PLA vs. PLLA and the literature results. (**a**) Yield strength, (**b**) ultimate tensile strength, (**c**) elastic modulus, (**d**) elongation at break.

The mechanical properties of stereocomplex PLA have previously been reported, although in a limited number of articles. As seen in Figure 12, the mechanical testing results of SC PLA were compared with the literature results. Korber investigated the mechanical properties of stereocomplex PLA extruded in a co-rotating twin-screw extruder (L/D = 40) with various nucleating agents and injection molded at different mold temperatures. Stereocomplex PLA extruded with 1% Finn nucleator produced the highest mechanical properties, as seen in Figure 12. They conclude that increasing mold temperature coincides with decreasing nucleation frequency and increasing crystal growth [38]. The results shown are when SC PLA with 1% Finn is molded at 33 °C. Su et al. studied the effect of different PDLA contents melt blended with PLLA in a rheometer to produce stereocomplex PLA. The results shown below in Figure 12 involve a 50/50 blend of hot-pressed stereocomplex PLA films. It was concluded that increasing the PDLA content led to increasing tensile strength and modulus, but decreasing elongation at break [34]. Samsuri et al. used stereocomplex PLA as a nucleating agent on extruded high molecular weight PLLA/PDLA blends and the results showed that SC PLA formation and crystalline fraction in the PLLA/PDLA blends coincided with the mechanical property improvements in the PLLA/PDLA blends. This is due to particle distribution, allowing for more initiation sites for stereocomplex formation and nucleation sites for improved crystallization rate [41].

Srithep et al. mixed PLLA and PDLA samples of varying percentages directly into an injection molding machine for processing compared to extrusion and solution blending. Interestingly, the mechanical properties decreased as the PDLA content increased [78]. This was most likely because not enough stereocomplex crystallites can form in the injection molding machine at their processing temperature (200 °C), and not enough shear mixing is taking place compared to an extruder. There is not enough shear mixing to distribute the PLLA and PDLA particles evenly during processing, leading to the decrease in the thermal and mechanical properties. To avoid this issue, Li et al. used branching agents of pyromelliticdianhydride (PMDA) and polyfunctional epoxy ether (PFE) to promote stereocomplex formation and long chain branching during single-screw extrusion (T = 180 °C). Both the tensile strength and modulus increased with branching agent concentration, and they were at their highest at 1.5% branching agent [79].

It is clear from Figure 12 that the produced SC PLA product is comparable or superior to that of the recent literature results. Compared to recent studies, this technique uses no additives or nucleators, and it is scaled up to a co-rotating twin-screw extruder (L/D = 40). In this study, the stereocomplex PLA product forms a highly oriented structure during extrusion stretching. It is cooled immediately after leaving the extruder in a cold-water bath, largely retaining the molecular orientation. This orientation results in a significant improvement in the mechanical properties. Similar studies have reported a similar behavior in other polymer blends and composites [80,81]. One report saw an improvement in the elastic response and oxygen barrier properties of polypropylene/polyamide blends due to the highly oriented structure that results [81]. Another report studied CO_2_ treated PLA/PTFE composites in which the researchers concluded that the tensile strength of the PLA composites correlated with an increase in the PTFE content, most likely due to the highly oriented *trans*-crystals [82]. This significant improvement in tensile behavior led us to propose stereocomplex PLA as melt spun fibers to reinforce PLA and other thermoplastics, particularly in textile applications.

### 3.6. Thermal Degradation via TGA

**Figure 13:**
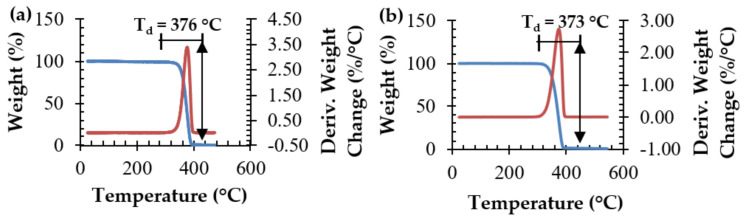
Thermal degradation of (**a**) stereocomplex PLA and (**b**) PLLA (L175).

The thermal decomposition temperature of SC PLA was determined and compared to commercial grade poly(L-lactide)—L175. From Figure 13a, stereocomplex PLA starts to decompose at ~297 °C, with a maximum thermal decomposition temperature of T_d_ ~376 °C; a percent weight loss of ~98% is depicted. No significant difference was seen compared to commercial PLLA (Figure 13b), which starts to decompose at ~293 °C and has a maximum thermal decomposition temperature of T_d_ ~373 °C; a percent weight loss of ~99% is depicted. This led us to conclude that while stereocomplexation can be attributed to an increase in the melting temperature, it does not necessarily contribute to improved resistance against thermal decomposition. The main factor affecting thermal degradation is most likely the molecular weight of the polymer. Since the PLLA (L175) analyzed here is the same PLLA polymer used in the production of stereocomplex PLA, the similarities in thermal degradation are understandable, as their molecular weights are assumed to be similar. It is hypothesized herein that the molecular weight of SC PLA is in fact a 50/50 ratio of the molecular weights of the PLLA and PDLA blended for stereocomplex PLA processing. To date, this has not been confirmed, as no suitable solvent has been found to dissolve stereocomplex PLA for molecular weight characterization (i.e., GPC); this is attributed to its high crystallinity thickness [8].

## 4. Conclusions

We developed a scalable, solventless method for the continuous manufacturing of stereocomplex PLA via the twin-screw extrusion of high molecular weight PLLA and PDLA. No additives or reagents were used in the process. The thermal characteristics/kinetics of stereocomplexation were first considered, and it was concluded that the process is temperature driven. Time did not play a role in the formation of stereocomplex PLA, as the process was almost instantaneous. The temperature was carefully considered to account for the solidification and thermal dissociation of the stereocomplex PLA. The processing conditions (temperature profile, screw configuration, etc.) were thus optimized based on these results, and 95% of the conversion of stereocomplex PLA was achieved according to the DSC results. The WAXD results indicate full stereocomplexation representing a triclinic unit cell, as opposed to PLLA and PDLA’s pseudo-orthorhombic crystal structure. 56–64% of total crystallinity was achieved for pure stereocomplex PLA, and a melting point of 240 °C was reported.

A significant improvement in the tensile properties were observed after stereocomplexation, particularly, the yield strength, tensile strength, modulus, toughness, and resilience. These results align with the increase in crystallinity and melting temperature as well as the high stereocomplex crystallite formation. We attributed this behavior to the intramolecular interactions taking place between PLLA and PDLA. In the extruder, the PLA chains are stretched to a highly oriented structure, allowing for promoted alignment of the chains. The structure is retained after immediate cooling, allowing for high crystallization.

These results lead us to propose stereocomplex PLA as a potential organic additive or fiber that can reinforce the crystallinity, thermal properties, and mechanical properties of neat PLA. The next steps should focus on manufacturing PLA/stereocomplex PLA molecular composites at various compositions to analyze its effect on the PLA material properties. Melt spun fibers of stereocomplex PLA will be manufactured for the composites. Injection molding of the composite tensile bars should be scaled up to the pilot scale to confirm the scalability of the process. The crystallization kinetics should be analyzed at various compositions and compared with talc under the same processing conditions. Additional screw configurations may also be explored in future studies. Finally, one may consider other processing techniques such as grafting of the stereocomplex PLA onto other materials to observe its effect on the material properties as opposed to physical blending. Stereocomplex PLA has commercial application as a value-added product for PLA and other polymers, particularly as melt-spun fibers in textile applications.

## Figures and Tables

**Table 2:** Material properties of the TotalEnergies Corbion standard grades of PLA.

Sample	o.p. (%)	σ_ult_ (MPa)	*E* (GPa)	T_g_ (^o^C)	T_pm_ (^o^C)	*X*_c_ (%)	M_n_ (kDa)	M_w_ (kDa)	PDI
L175	99.68	50	3.5	63.13	175.10	47.52	103	172	1.67
D120	99.81	50	3.5	62.21	177.52	51.40	92	150	1.64

**Table 3:** The DSC results on the thermal properties and crystallinity of stereocomplex PLA at various collection times.

Collection Time (min)	*f_sc_* (%)	*X_c_* (%)	T_pm,sc_ (°C)	T_pm,hc_ (°C)	T_g_ (°C)
5	85	64	241	178	63
10	92	61	240	175	64
20	93	62	240	175	65
40	95	56	240	175	61

**Table 4:** The mechanical properties of injection molded neat PLLA vs. SC PLA.

Name	σ_break_ (MPa)	ε_break_ (%)	σ_y_ (MPa)	σ_ult_ (MPa)	*E_elastic_* (GPa)	ε_p_ (%)	*U_T_* (J/m^3^)	*U_R_* (MPa)
PLLA	36.94 ± 1.5	9.62 ± 0.6	36.73 ± 1.3	48.67 ± 2.3	1.52 ± 0.07	7.18 ± 0.10	3460.17 ± 50.7	0.44 ± 0.02
SC PLA	96.29 ± 2.6	9.98 ± 1.2	70.87 ± 2.5	106.34 ± 3.1	2.93 ± 0.1	6.69 ± 0.08	8107.45 ± 65.3	0.86 ± 0.05

## Data Availability

The data presented in this study are available in this article “Scalable Continuous Manufacturing Process of Stereocomplex PLA via Twin-Screw Extrusion”.
